# Parents’ and Caregivers’ Experiences of Procedural Holding and Restraint: ‘We Want To Be Their Safe Place and Not Associated With Medical Trauma’: A Qualitative Descriptive Study

**DOI:** 10.1111/jocn.70462

**Published:** 2026-07-24

**Authors:** Lucy Bray, Julie Blamires, Michael Neufeld, Jenny O‘neill, Blake Peck, Erin Hargreaves, Sharon Kinney, Sage Hay

**Affiliations:** ^1^ School of Nursing, Midwifery and Allied Health, Faculty of Health, Social Care and Medicine Edge Hill University Ormskirk UK; ^2^ School of Nursing Auckland University of Technology Auckland New Zealand; ^3^ Department of Nursing Research The Royal Children‘s Hospital Melbourne Victoria Australia; ^4^ Department of Nursing, Melbourne School of Health Sciences The University of Melbourne Melbourne Victoria Australia; ^5^ School of Nursing and Midwifery Deakin University Burwood Victoria Australia

## Abstract

**Aim:**

To explore parent/caregivers’ perceptions of procedures in which their child was held still and the reported impact of these experiences on both the child and parent.

**Design:**

Qualitative descriptive study using an online survey.

**Methods:**

Three research teams (United Kingdom (UK), New Zealand (NZ), and Australia) administered a qualitative survey to parents/caregivers of a child who had been held for a procedure in the last 2 years. Data were analysed using inductive thematic analysis.

**Results:**

One hundred and twenty parents and three caregivers described 215 clinical procedures during which their child had been held. Four themes, conceptualised as procedural journeys, captured how parents described experiences unfolding over time: (1) Unravelling restraint, (2) Inevitable restraint led by professionals, (3) Inevitable restraint led by parents/caregivers, and (4) Child‐centred holding. Parent/caregivers'accounts highlighted how procedures characterised by limited planning, rapid escalation, reduced responsiveness to child distress and restraint were experienced as particularly challenging and linked to feelings of guilt, regret and trauma. Procedures involving preparation, communication, flexibility and supportive comfort holds were described more positively and as less traumatic.

**Conclusion:**

The use of restraint was described as distressing for both the child and the parent/caregiver, particularly when procedures unfolded without adequate planning. Approaches that prioritised preparation and collaboration were perceived to support more positive experiences.

**Implications for the Profession and/or Patient Care:**

There is a need to move away from default or reactive restraint practices towards more deliberate, rights‐based, trauma‐informed procedural care.

**Reporting Method:**

The Consolidated Criteria for Reporting Qualitative Research (COREQ) were utilised when reporting findings.

**Patient or Public Contribution:**

To ensure that study materials and survey questions were clear and relevant, consultation occurred with two parents in the UK and three parents in NZ. The study design and questions were also presented to the New Zealand Mātauranga Māori Committee for feedback to ensure cultural appropriateness.

## What Does This Paper Contribute to the Wider Global Clinical Community?

- Poorly managed procedures involving child distress and the use of default or reactive restraint can result in parents experiencing guilt, regret, and trauma
- A pause to plan a procedure can mitigate against the use of restraint as a ‘preferred’ approach and support parent‘s meaningful involvement in their child‘s procedure.
- Positive procedural experiences align with an increased recognition of trauma‐informed, rights‐based approaches in paediatric clinical care.

## 1 Introduction

Children and young people regularly attend healthcare settings such as general practice clinics, public health services, dental clinics and hospitals, where they commonly undergo clinical procedures. In some cases, children become anxious, upset, distressed or resistant before or during a procedure. Some of these procedures involve a child being held by a parent, caregiver or healthcare professional to keep them still or safe while the procedure is completed. Holding in this context could involve a parent or caregiver giving their child a supportive hug while the child sits on their lap, or, in some instances, it might involve parents and healthcare professionals restraining a child (Bray et al. 2016).

## 2 Background

The practice of restraining or holding a child during a clinical procedure is a common feature of paediatric healthcare (de Souza et al. 2026). There is a growing body of evidence that suggests these holds can be very distressing for some children (Forsner et al. 2023; Snodin et al. 2026), their parents (Hay et al. 2025), and healthcare professionals (Svendsen et al. 2017). Terminology used within paediatric practice to define the act of holding a child while a procedure is completed is inconsistent and contested (da Silva et al. 2025). The multitude of terms used includes clinical holding, therapeutic holding, supportive holding, comfort positioning, restrictive physical intervention and restraint (Coyne and Scott 2014; Østberg et al. 2024). Despite changes in terminology over time, the practice of physically holding a child to facilitate the completion of a clinical procedure remains unchanged (Bray et al. 2018; Page et al. 2015). Recent international work to develop rights‐based standards for children having procedures defines a restraining hold as ‘any action to prevent a child moving freely against their choice or will while expressing signs of refusal’ and a supportive hold as one where a child ‘agrees to the procedure and positioning and/or does not express signs of refusal’ (iSupport, n.d., p. 4).

Justifications from healthcare professionals for the use of restraining holds include: (1) to ensure completion of the procedure, (2) to prevent injury to the child or healthcare professional and (3) to minimise procedural delays (Cummings 2015). Persistent myths continue to shape some procedural practices, such as the belief that children are too young to comprehend what is happening, or that procedures are best completed as quickly as possible without engagement or preparation (Bray et al. 2016). Throughout the literature, it is indicated that younger children (Brenner et al. 2015), those with additional needs (Murdoch and Chang 2022), and those displaying distress (Bray et al. 2016) are the most likely to be held. Despite evidence to show the positive impact that distraction techniques, parental presence and comforting strategies can have on a child's procedural experience, the use of holding and restraint to complete a procedure is still an accepted and often unchallenged part of paediatric practice (Bray et al. 2018; Coyne and Scott 2014).

Many parents or caregivers will hold their child for a procedure, such as an immunisation, examination or treatment. Parents often rightfully feel that their presence is crucial throughout the procedure and that holding their child provides support and brings comfort to their child (Hay et al. 2025; Karlsson et al. 2014). Evidence shows that a child is less likely to be distressed if their parent is present (Rheel et al. 2022), and if they are held in a comforting, supportive position by their parent, rather than positioned or restrained in a supine position (Sparks et al. 2007; Petkus et al. 2025; Park et al. 2024). However, some parents are surprised and feel uncomfortable when their child is held for a procedure (Brenner 2013) and may find it difficult and upsetting if forceful holding is used (McGrath et al. 2002). In previous literature, parents have also described feelings of letting their child down (Karlsson et al. 2014). Whilst evidence shows the significant role parents have in their child's procedure, there is a lack of research focusing explicitly on parent and caregivers’ experiences of their child being held for a procedure. There is also a gap in understanding the reported short‐ and long‐term impacts of this holding and restraint on parents or caregivers and their child.

## 3 The Study

### 3.1 Aim

This international study aimed to explore parents’ and caregivers’ perceptions of procedures in which their child was held still and the reported impact of these experiences on both the child and the parent.

## 4 Methods

### 4.1 Design

This study employed a qualitative descriptive design using an online survey (Sandelowski 2000), to explore parents’ and caregivers’ experiences and perceptions of their child being held during a clinical procedure. This approach was particularly suited to the aim of the study, as it allows for a detailed account of participants’ experiences while remaining grounded in their own language and perspectives. Qualitative description is particularly suited to examining the ‘who, what and where’ of events while also capturing how parents and caregivers make sense of their experiences and the impact of these events on themselves and their child (Kim et al. 2017). The study was conducted using an inductive analytic approach and was not guided or underpinned by a pre‐existing framework. Instead, findings were grounded in participants’ accounts, allowing themes to be developed directly from the data.

A qualitative survey method was employed, which is increasingly recognised as an effective approach for gathering rich narrative accounts from a broad group of participants (Braun et al. 2021). Qualitative surveys invite participants to respond to open‐ended questions in their own words, generating textual data that foreground participants’ perspectives without imposing restrictive response categories (Braun et al. 2021). This method allows researchers to explore experiences in depth while also identifying patterns across a larger and potentially more diverse sample than is typical in interview‐based designs. The multi‐country design enabled exploration of parents’ and caregivers’ experiences across different healthcare contexts, enhancing the breadth and relevance of the findings.

### 4.2 Public Involvement

The study team consulted with two parents in the UK whose child had undergone a procedure to ensure that study materials were accessible and clear and that survey questions were relevant and appropriate. Similar consultation was undertaken with three parent advisors in NZ, with their feedback used to further refine survey questions and develop consistent prompts. Following Te Ara Tika guidelines, the study design and draft survey questions were also presented to the New Zealand Mātauranga Māori Committee (Health Research Council of New Zealand 2010). This consultation ensured that the study design, research approach, recruitment methods and participant information sheets were culturally appropriate within a NZ context. Based on feedback from these consultations, a clearer definition of what was meant by holding was included within the survey, and instructional text prompts were added to each question. A question was also added to the end of the survey to enable parents to share any additional information they felt was important. While a formal pilot study was not undertaken, consultation with parent advisors served to refine and improve the clarity and relevance of the survey questions prior to data collection.

### 4.3 Sample

Participants were parents or caregivers whose child (aged 0–18 years) had experienced holding as part of a clinical procedure in the last 2 years. A procedure was defined within the study as any healthcare test, treatment or examination and instances of holding or restraint linked to mental health admission were excluded. These criteria were defined to focus on procedural holding within a general healthcare context, as restraint related to mental health admission represents a distinct clinical context.

Convenience sampling enabled insights to be gained from a wide range of participants in line with the exploratory nature of the qualitative descriptive design (Doyle et al. 2020). Sample adequacy was guided by the principle of information power, whereby the required sample size is determined by the relevance, richness, and specificity of the data in relation to the study aim, rather than participant numbers alone (Malterud et al. 2016). In this study, the focused aim, combined with rich experiential accounts and consistent patterns identified across the large and diverse dataset, indicated that sufficient information power had been achieved. No predefined sample size was set, with recruitment guided instead by the achievement of sufficient information power.

### 4.4 Recruitment and Setting

Within the UK and NZ, the study was advertised by circulation of a flyer through established personal and professional healthcare networks such as social media accounts and links with charities and parent support groups. The online survey was accessible through a QR code and link on the flyer. Within Australia, recruitment occurred within a single tertiary paediatric hospital. Parents and caregivers were approached by members of the Australian research team and provided with a flyer that contained a QR code linking to the study information and survey. Parents could choose to complete the survey immediately on their phone, use a study iPad or complete it later. Differences in recruitment approaches reflected local feasibility and access to participants.

### 4.5 Data Collection

The online survey was collaboratively developed by the international research team across the UK, NZ and Australia, bringing together clinical and academic expertise in paediatric care and qualitative research. The survey started with a brief overview of participation and included a tick box for parents/caregivers to confirm they had read the study information and provided consent to take part. The survey was confidential, with no identifying information required from participants, and asked for minimal demographic information including country of residence, parent/caregiver role, their child's identified gender, and if their child had any long‐term health conditions, disabilities or neurodivergence. These data were collected to help characterise the sample and provide contextual information about participants and their child, rather than to inform any analytic interpretation. The two open text questions asked participants to describe two ‘tests, treatments or examinations’ where their child had been held (either by themselves, another family member, and/or a staff member). Responses to questions were not mandatory, allowing participants to provide as much or as little information as they wanted, although prompts were provided, as shown in Table 1, to guide participants to include as much information as possible in their response. Where participants included potentially identifiable details (e.g., names or locations) within open‐text responses, these details were removed during data cleaning prior to analysis.

**TABLE 1:** Prompts to guide the open‐text responses.

How old was your child when they had this procedure?
What test, treatment or examination were they having?
What happened? (Who was there? Was your child upset or calm? Who held them, and how?)
What went well, and what did not go so well?
What could have been done differently?
What has been the impact of this experience on you and your child?

The survey was administered in NZ and Australia using the platform REDCap and, in the UK, using SurveyMonkey. Data were collected between November 2024 and March 2025.

### 4.6 Rigour and Reflexivity

Rigour of this study was carefully considered given the novel method of data collection for a qualitative study. As a survey does not allow for close or prolonged engagement with the participant, care was taken to promote the credibility of the findings by staying close to the participants' accounts during analysis and ensuring a clear audit trail of analytical decision‐making. The study was undertaken by an international team of researchers, consisting of paediatric nurses, child and family health academics, a nurse ethicist and an undergraduate medical student. Collectively, the team was experienced in qualitative research with six PhD‐trained researchers and one Master's‐prepared researcher. The team was reflexive throughout the study, engaging in structured discussions at each analytical meeting to surface and interrogate the potential influence of their positionality on data interpretation, recognising the influence of their roles as nurses and parents and of their previous investigations within the field. Five members of the research team are parents, and two members of the research team work in clinical roles within a tertiary paediatric hospital.

### 4.7 Data Analysis

The qualitative data were analysed using inductive thematic analysis according to the six steps identified by Braun and Clarke (2006). Analysis was an iterative and flexible process, with codes and themes derived from the data. Each in‐country team, consisting of two to three team members, independently coded their data. These initial codes were then shared and discussed within multiple analytical meetings; six international team members were present on each occasion. This collaborative structure was intentional. Because of the multi‐country design of the study, it was important to ensure that codes and developing themes were examined across cultural and clinical contexts and were not unduly shaped by any single country's data. These discussions resulted in some analytical directions being challenged, and discrepancies or uncertainties were acknowledged and debated. An initial descriptive analysis of parents’ and caregivers’ accounts was undertaken to identify patterns in experiences and their perceived impact. As analysis progressed, it became evident that parents consistently described their child's procedural experience as an unfolding sequence of events, including factors occurring before, during, and after the procedure. This temporal structure of accounts was central to how parents and caregivers described and made sense of holding and restraint. To retain this experiential and process‐oriented dimension, themes were developed as procedural journeys, capturing both the sequence of events and how parents interpreted and responded to them in ways that shaped the overall experience.

### 4.8 Ethical Considerations

Ethical approval was sought within each participating country: UK [ETH‐2425‐001], NZ [AUTEC 24/240] and Australia [HREC/111793/RCHM‐2024]. In Australia, this included approval for recruitment at the tertiary paediatric hospital where data collection occurred. Participants were provided with clear information at the beginning of the survey regarding what participation entailed, that participation was completely voluntary and that, as responses were anonymous, participants would not be able to change or withdraw their responses once their survey was submitted. Consent was obtained by participants ticking a box at the beginning of the survey. In recognition that parents/caregivers reflecting on difficult experiences with their child might become upset, links to country‐specific support organisations were provided at the end of the survey. Due to anonymity of the responses, participants could not provide feedback on findings.

## 5 Findings

### 5.1 Participants

In total, 124 complete survey responses were received (121 parents and three caregivers). Fifty participants were from Australia, 42 from NZ, 29 from the UK and three from other countries (the Netherlands, Sweden and the United States of America), who responded to the UK survey after accessing it through the social media flyer. These three responses are reported within the UK dataset. Participant characteristics specific to each country are detailed in Table 2. As recruitment was conducted via convenience sampling, including through social media, it was not possible to determine the number of individuals who viewed the survey but did not complete it.

**TABLE 2:** Participant characteristics according to the three countries tha led data collection.

Participant characteristics	UK	NZ	Australia
Number of parent/caregiver responses	32	42	50
Number of procedures described	52	93	70
Age of child at time of procedure	5 years (mean) range 2 weeks—14 years	3.7 years (mean) range 1 month–15 years	3.7 years (mean) range 1 month—12 years
Caregiver role	2 Mothers, 3 Fathers	38 Mothers, 2 Fathers 1 Grandparent 1 Aunt	39 Mothers, 10 Fathers 1 Grandparent
Child‘s identified gender (as reported by parent/caregiver)	11 Male 19 Female 2 Not shared	21 Male 18 Female 1 Non‐binary 2 Not shared	22 Male 28 Female
Ethnicity of parent or caregiver (open text response)	28 White 2 South Asian 2 Not shared	28 European/Pakeha 2 Maori 2 MELAA 2 Pasifika 2 Asian 4 ‘Other’ 2 Not shared	35 Australian 1 New Zealander 1 South Asian 5 East/South‐East Asian 4 Middle Eastern 4 European
Child neurodivergence, long‐term medical condition or disability (as reported by parent or caregiver)	4 Intellectual disability 7 Neurodivergent 10 Long‐term medical condition	3 Intellectual disability 9 Neurodivergent (incl. 2 possible/querying) 17 Long‐term medical condition 2 Mental health condition	5 Intellectual disability 8 Neurodivergent 15 Long‐term medical condition

Parents and caregivers across countries described a range of clinical procedures, including needle‐related procedures, medical imaging, feeding tube insertion, anaesthetic induction, dressing changes, medical examinations, pathology swabs, administration of oral medication and measurement of vital signs. Notably, the most frequently reported procedure was a blood test.

### 5.2 Themes

Four themes illustrate distinct procedural journeys described by parents and caregivers. These journeys reflect how parents/caregivers narrated their child's procedural experience over time, often beginning prior to the procedure, describing the escalation or management of distress during the event and reflecting on its impact afterwards. This temporal and experiential framing was retained in the analysis, as it was central to how parents/caregivers made sense of holding and restraint and what shaped positive or negative experiences. These journeys therefore represent not predefined classifications but parents'/caregivers' interpretations of how events unfolded over time and what shaped their experiences. The four procedural journeys identified were: (1) Unravelling restraint, (2) Inevitable restraint led by professionals, (3) Inevitable restraint led by parents and (4) Child‐centred holding. Supporting quotes are represented by the country, participant number, and age of the child at time of procedure.

Parents/caregivers descriptions of the four procedural journeys are shown in Figure 1, including the described emotional state of the child and parent and the short‐ and long‐term impacts of the procedural experience. These journeys are not presented as hierarchical or as representing distinctions between positive and negative experiences. However, as the journeys move from left to right, parents/caregivers described less child distress and reduced child and parent trauma and harm.

**FIGURE 1:**
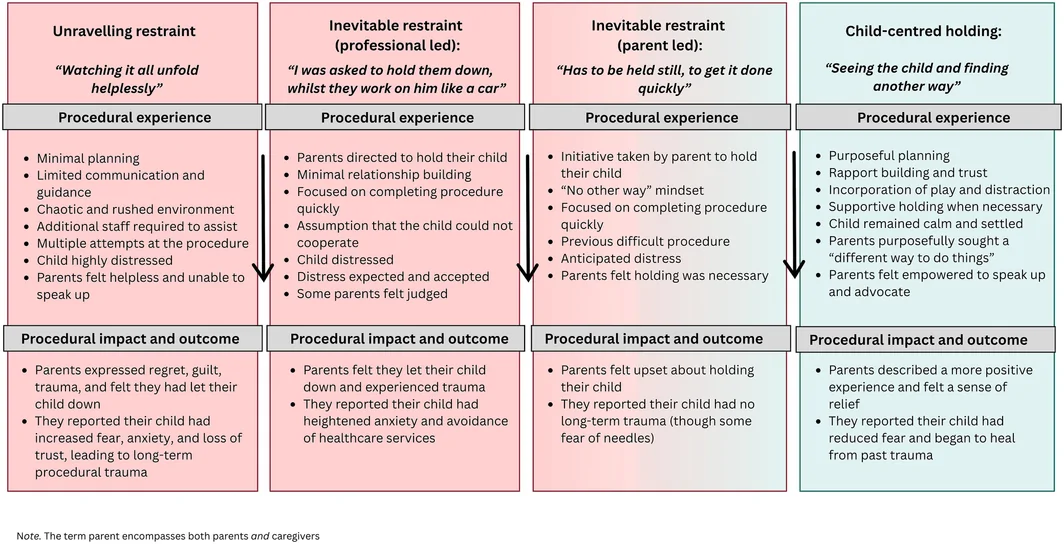
Overview of procedural journeys. [Colour figure can be viewed at wileyonlinelibrary.com]

### 5.3 Unravelling Restraint: ‘Watching it All Unfold Helplessly’

Within this procedural journey, parents and caregivers described experiences in which restraint was used to keep their child still, often accompanied by high levels of distress, with parents and caregivers using words such as ‘kicking, screaming’ (NZ, P35, 5‐year‐old), ‘distraught’ and ‘hysterical’ (UK, P24, 3‐year‐old). These journey descriptions were characterised by situations where children were already distressed before the procedure began or became distressed as it started, with distress often escalating as the procedure progressed.

He entered unsure and he escalated quickly and left with a huge fear. It was an awful experience. He was scared and strong and fighting me throughout as well as crying hysterically. (Australia, P42, 5‐year‐old, neurodivergent)

Parents and caregivers described these experiences as ‘more panicked, more rushed, less listening, less taking time’ and often situations where ‘more nurses were brought into the room’ (UK, P26, 6‐year‐old). Across all countries in this study, parents and caregivers described accounts of multiple people restraining their child during a procedure, ‘I tried to hold him, but couldn‘t hold him still, so the nurse had to call for two colleagues, who held his legs. He was very upset. Kicking and wiggling’ (UK, P32, 12‐year‐old, neurodivergent and intellectual disability). ‘There were two nurses, me, dad and two doctors He was upset and fighting all of us’ (NZ, P40, 17‐month‐old, long‐term medical condition and intellectual disability). The following excerpt involved healthcare professionals and parents restraining a young person with an intellectual disability:

Last time she had her bloods done, four of us had to hold her down and she was extremely distraught and lashing out to be let go of. She cried and screamed and I had to hold onto her very tight. (UK, P21, 14‐year‐old)

Some parents and caregivers described restraining their child, despite knowing it was causing trauma, due to their perception that ‘if you said ‘no’ as a parent or caregiver you would be noted down as being non‐complaint’ (UK, P14, 3‐year‐old). One mother described being powerless to speak up as she ‘watched on helplessly. I was naive at the time and didn‘t realise I could and should have stepped in to stop it (UK, P24, 2‐year‐old). Another mother had tried to raise a concern about the use of restraint but ‘was made to feel like I was whistleblowing and it took several attempts to get someone to listen’ (UK, P22, 5‐year‐old).

Within this procedural journey, parents described limited, poor or no communication with their child: ‘The staff didn‘t give two shits about him or his needs; they didn‘t even look at him’ (NZ, P34, 8‐year‐old, neurodivergent). These procedures were also characterised by parents and caregivers describing being physically excluded: ‘I was told to stay out of the room’ (Australia, P16, 18‐month‐old) or feeling emotionally separated as an onlooker to unravelling restraint. Parents and caregivers reported these journeys as ‘a traumatic experience for my child’ (NZ, P35, 5‐year‐old), resulting in negative consequences where their child now has ‘a huge fear of the hospital and doctors’ (UK, P24, 2‐year‐old), struggled to ‘be touched by doctors at routine visits’ (NZ, P28, 2‐year‐old, neurodivergent), and ‘refuses to get out of the car as she automatically associates the hospital with bloods’ (UK, P21, 14‐year‐old, intellectual disability).

In some cases, their child had not been able to attend the healthcare services or ‘have any more tests done since’ (NZ, P35, 5‐year‐old) and some were ‘still struggling years later’ (Australia, P35, 8‐year‐old). In addition to children being reported as experiencing long‐term harm from these difficult procedural experiences, parents and caregivers also described living with ‘nightmares about the experience’ (NZ, P28, 2‐year‐old), ‘regret [about] not advocating more strongly’ (Australia, P16, 18‐month‐old), and reported feelings of shame and inadequacy for not intervening to keep my kids safe and secure’ (NZ, P3, 34‐week‐old, twins).

### 5.4 Inevitable Restraint Led by Professionals: ‘I Was Asked to Hold Them Down, Whilst They Work on Him Like a Car’

In this procedural journey, parents and caregivers described experiences in which restraint was initiated or directed by healthcare professionals. Parents and caregivers described how they had been ‘instructed to hold her hands down’ (UK, P19, 5‐year‐old) or asked, ‘to hold my child on my lap and hold his arms still’ (Australia, P3, 3‐month‐old), and one mother explained how ‘the doctor asked me to hold her down tightly, lying flat on her back’ (UK, P8, 2‐week‐old).

Some professionals were described as directing parents and caregivers to ‘just hold him tighter’ (NZ, P27, 5‐year‐old) to keep their child still, or physically positioning a parent to more effectively restrain their child: ‘The nurse kept positioning me so my arms were pinning my daughter down and holding her other arm tightly away’(UK, P23, 5‐year‐old). In some cases, parents and caregivers described how ‘there didn‘t seem to be any other option’ (UK, P25, 2‐year‐old) and professionals had ‘automatically assumed’ (UK, P18, 5‐year‐old) that parents and caregivers would hold or restrain their child. Parents and caregivers described this as creating a tension between their perceived role as comforter and protector of their child, and their perceived obligation to enable the procedure to be completed:

They expected us as parents to restrain her. This was very frustrating, since as a parent you are trying to protect and comfort your child, but in this situation, you can‘t do that. So you experience a role conflict. It made us feel very powerless. She was very upset and panicking. I felt horrible. (UK, P28, 5‐year‐old, long‐term medical condition)

If parents and caregivers became upset, they described being ‘told I was too sensitive; it needed to be done that way!’ (NZ, P3, 34‐week‐old, twins). A child‘s emotional distress was described as expected by health professionals and unavoidable, particularly if the child had an intellectual disability or was neurodivergent. Professionals were described as not communicating with their child, ‘the worst is when doctors only speak to me and ignore him like they‘re working on a car’ (UK, P29, 4‐year‐old, intellectual disability) or having ‘no understanding of how to manage and treat the fear, holding the child down won‘t help the feelings they feel’ (Australia, P21, age unknown, neurodivergent).

Parents and caregivers described how these experiences shaped their child‘s ongoing relationship with healthcare, often resulting in anticipatory anxiety and avoidance. One parent described their child ‘is scared when he sees doctors and nurses’ (Australia, P1, 4‐year‐old). In some cases, these experiences also affected the parent–child relationship, with parents and caregivers describing a perceived loss of trust. Parents described feeling that they had let their child down or contributed to their distress, for example, ‘she was traumatised and so upset that I let them hurt her’ (UK, P24, 3‐year‐old). Parents and caregivers also described longer‐term impacts on both the child and them following these experiences.

This is when the PTSD [post‐traumatic syndrome disorder] of my daughter spiraled out of control. She needed EMDR [Eye Movement Desensitization and Reprocessing therapy] and psychological interventions to recover from that. I had EMDR too, because I suffered from PTSD as well as a result of all the procedures with restraint. (UK, P28, 5‐year‐old, long‐term medical condition)

### 5.5 Inevitable Restraint Led by Parents and Caregivers: ‘Has to be Held Still, to Get it Done Quickly’

Within this procedural journey, parents and caregivers described experiences in which they directed, initiated, or undertook holding and restraint themselves to complete the procedure. In these accounts, parents and caregivers commonly used words such as ‘had to’ (Australia, P27, 6‐year‐old), ‘it‘s what‘s required’ (NZ, P15, 12‐year‐old, neurodivergent and mental health condition), and ‘he needs to be held’ (Australia, P1, 4‐year‐old). One mother detailed her experience of holding her child for vaccinations:

My husband held him, and I held his arm. He screamed and wriggled the whole time. The nurse was very supportive, but I was scared too. I wish he wasn‘t held. He had bruises on his arms. He [the child] was also very angry. I think we needed to give him time but it was also very hard because we came to the hospital and didn‘t want to go home without it [vaccination]. So it must be done. (Australia, P26, 6‐year‐old)

In many of these accounts, parents and caregivers described choosing to hold and restrain their child ‘I can easily restrain my daughter and would not want anyone else to restrain her’ (UK, P4, 12‐year‐old, neurodivergent and intellectual disability), and ‘I feel confident that I have a good technique for holding flailing limbs’ (NZ, P4, 15‐month‐old), even when the descriptions of holding were difficult:

He is always held at dental appointments, he has never sat in the dentist seat yet and my husband usually holds him, whilst I try to prise his mouth open enough for the dentist to look. (UK, P26, 8‐year‐old, neurodivergent)

Some accounts described how previous difficult procedural experiences, where children were distressed and restrained, shaped parents' and caregivers' expectations and decision‐making. These experiences led some to assume that restraint would be required and to plan accordingly, for example, ‘we both went so he [father] could hold/restrain her while being examined’ (UK, P31, 9‐year‐old, neurodivergent). Parents and caregivers of these children, many of whom were neurodivergent or had intellectual disabilities, described being frustrated when professionals attempted a different approach:

My child has to be held still for many tests and checks and procedures, as he does not comply. Teeth check ups, bloods, routine examinations, scans, check throat/tonsils, anaesthesia prior to surgery, placing equipment for a sleep study, weight. The best thing to do is to do this quickly and listen to me for advice on how to do this with minimal distress. The staff keep trying to encourage him to comply but the longer they do this, the more he will become distressed and worry about the event. (UK, P27, 10‐year‐old, neurodivergent with complex needs)

For many parents and caregivers, restraint was framed as a pragmatic and protective decision, with some reflecting that the quick use of restraint to complete the procedure was the best approach and that ‘not much could have been done differently’ (UK, P31, 10‐year‐old, neurodivergent). However, this was often accompanied by underlying uncertainty or concern about longer‐term implications, as despite this parent reflecting that their daughter ‘doesn‘t remember the examination’ they ‘worry about the future, and how we are going to manage appointments when she gets beyond the age when she can be held’ (UK, P31, 10‐year‐old, neurodivergent).

### 5.6 Child‐Centred Holding: ‘Seeing the Child and Finding An‐other Way’

A contrasting procedural journey described by parents and caregivers was characterised by elements aligning with a child‐centred approach, either from the outset or emerging in response to the child‘s distress, prompting a pause to ‘find another way’ (UK, P8, 2‐year‐old). Parents and caregivers described these approaches as recognising the child‘s agency and capacity, as ‘kids are smart and capable, and we need to start giving them credit for that, instead of assuming they won‘t cooperate and treating them like wild animals’ (UK, P23, 6‐year‐old). In many cases, a child‐centred approach was led by healthcare professionals:

My daughter needed her 15‐month immunisations. They were administered by a nurse we had never met before. She provided me with a toy to distract my child and was well prepared and confident. She chatted and played with my child and told her what was going to happen. I held my child in my lap and the nurse held the leg she was going to inject into. My daughter cried briefly but the nurse clapped her hands and smiled and distracted my daughter with stickers. This was a positive experience for both my daughter and I. (NZ, P41, 15‐month‐old)

In some cases, the child‐centred approach was also led by parents and caregivers, who felt compelled to advocate for their child after a previous difficult procedural experience: ‘We no longer hold her for treatment or procedures. Sometimes that means going slower than professionals would like, but I now listen to her’ (NZ, P12, 3‐year‐old, long‐term medical condition).

Parents and caregivers reflected that ‘these experiences were by far less traumatic’ (NZ, P28, 2‐year‐old) and helped their child feel ‘happy because she felt heard’ (NZ, P39, 9‐year‐old), and stay really calm, as it‘s all over before there‘s any distress’ (UK, P23, 6‐year‐old). Child‐centred procedures were characterised by ‘waiting for him to feel ready for the procedure’ (Australia, P1, 4‐year‐old), using ‘friendly’ sing‐song voices, stickers and not waiting a long time’ (Australia, P35, 3‐year‐old), ‘talking to my daughter about her preference’ (UK, P18, 5‐year‐old), and ‘having access to some sensory toys that can distract the thought process’ (Australia, P21, age unknown, neurodivergent).

Within this procedural journey, parents and caregivers also described actively disrupting the expected use of holding and restraint by challenging professionals to adopt a different, more child‐centred approach: ‘I asked to try my way’ (NZ, P26, 16‐month‐old, long‐term medical condition).

It helped that the doctor didn‘t pressure us or rush us, we were able to change tactics to find something that worked, rather than persevering whilst she was upset. It helped that the staff didn‘t try to force or manhandle our child and let us lead the way and come up with a solution. (UK, P15, 1‐year‐old)

Challenging professionals to pause and ‘re‐think’ a procedure was described by parents and caregivers as ‘the hard part’ and they reflected that many parents and caregivers ‘don‘t know that they can say ‘stop’ or ‘no’ if the child‘s boundaries are crossed’ (UK, P30, 8‐year‐old, long‐term medical condition). Some parents and caregivers described a developing sense of confidence in advocating for their child with one parent describing the moment when her child was distressed and she had the ‘eventual realisation that I had the power to stop this’ (UK, P22, 6‐year‐old).

Parents and caregivers reflected that adopting child‐centred approaches made them feel ‘quite overwhelmed with gratitude for the staff’ (UK, P15, 1‐year‐old) and they described that it was an important ‘investment to make procedures less traumatic for kids, less traumatic for parents and caregivers, and makes kids more willing to engage in healthcare in the future’ (UK, P23, 6‐year‐old) and overall is ‘better than holding kids down’ (Australia, P21, age unknown, neurodivergent).

## 6 Discussion

This is the first international paper that has explored parent and caregivers' reports of their child being held and restrained for a clinical procedure and the short‐ and long‐term impact described as resulting from these experiences. The procedural journeys identified in this study highlight how parents and caregivers experience and make sense of these events as unfolding over time, with distressing encounters shaping both immediate and longer‐term emotional responses. Parents and caregivers described how distressing experiences could lead to feelings of helplessness and longer‐term feelings of regret, guilt, and, in some cases, trauma. In addition to their own experiences, parents and caregivers reflected on how these encounters involving distress and restraint appeared to influence their child's response to healthcare, including increased fear, anxiety, and reluctance to re‐engage with healthcare services.

Parents and caregivers described procedural journeys with limited or absent planning and rapidly escalating distress as particularly difficult. Within these experiences, parents and caregivers reported feeling out of control, helpless, and obligated to adopt the role of restraining their child during the procedure. These accounts highlight how the pace and unfolding of events shaped parents' and caregivers' ability to engage, respond, and advocate for their child. These findings align with existing literature suggesting that lack of planning amongst healthcare professionals performing a procedure (Bray et al. 2018), and pressures related to time and institutional circumstances (Sahlberg et al. 2020), can contribute to reactive approaches where restraint may be more likely to occur (Forsner et al. 2023). Experiences of participants in our study suggested that they had not been prepared for the rapid escalation and their involvement in the procedure. Elsewhere it has been shown that healthcare professionals expect that parents and caregivers will be present during their child's procedure and it is almost taken for granted that parents and caregivers will assist in the restraint of their child (Svendsen et al. 2017). The role of parents and caregivers restraining their child as an ‘enforcer’ (Hay et al. 2025) often unfolds without agreement or purposeful planning.

Parent and caregivers' accounts within the procedural journeys highlight how distressing procedural experiences can lead to feeling out of control and lasting feelings of regret and guilt. Consistent with previous research, parents' descriptions of limited control, upset, and feeling they had let their child down reflect recognised associations with distress and post‐traumatic stress responses in healthcare contexts (Ari et al. 2018; UK Trauma Council 2025). The accounts also highlight how repeated negative experiences within healthcare services may contribute to an accumulation of trauma over time for both children and their parents. Evidence has shown that children admitted to intensive care and their parents and caregivers (de Pellegars et al. 2024), children undergoing surgery (Stanzel and Sierau 2022), and children who have had cancer treatment (Al‐Saadi et al. 2022) can experience post‐traumatic stress disorder symptoms and how repeated distressing medical procedures performed in infancy can cause long‐term trauma and dissociation into adolescence (Diseth 2006). However, less is written about the short‐ and long‐term reported impact of a distressing minor procedure such as an ultrasound, X‐ray, vaccination, or blood tests for children and their parent and caregiver.

Parents' and caregivers' descriptions in the current study show that there can be missed opportunities to acknowledge and address the distress associated with difficult procedures, leaving parents and caregivers to deal with and make sense of what has been described in the literature as the emotional ‘debris’ after the use of restraint (Neilson et al. 2025). The lack of formal debriefs or discussion can also result in healthcare professionals and organisations missing opportunities to reflect on and think ‘what could have been done differently to help inform the next procedure.’

Of particular interest is how parents and caregivers described the difficulty and distress they faced in supporting their child who was neurodivergent and/or had an intellectual disability while having a procedure. As the findings show, in some cases restraint was assumed necessary from the outset to complete the procedure. The assumptions described by some parents and caregivers that a quick procedure involving restraint was in the best interests or the ‘only way’ for their child with neurodivergence and/or intellectual disability, limits dialogue and reflection of whether alternative approaches may result in less distress. Within these procedural journeys, restraint was at times described as a default ‘go‐to’ response rather than as a last resort, as recommended within most professional guidance. Healthcare professionals often rely on parents of children with intellectual disabilities to support care (Oulton et al. 2024), and while recognising and valuing this expertise is important, parents' accounts in this study suggest this may also reduce opportunities to re‐evaluate procedural options.

The experiences of parents and caregivers in the current study suggest how clear planning and preparation can support positive procedural journeys. In these instances, parents and caregivers felt empowered and supported to advocate for their child, and healthcare professionals were described as pausing to discuss and plan a procedure and were open to further pauses in the event the child became distressed. This is consistent with existing research suggesting there are better outcomes when healthcare professionals develop a plan, prepare children, and engage in a ‘clinical pause’ (Bray et al. 2019, 168) prior to a procedure, allowing time to consider the child's preferences and alternative approaches. Consistent with the literature, parents and caregivers in our study described information about what to expect, involvement of play therapy or other distraction techniques, and a “heads‐up” about potential for the parent to be asked to adopt a supportive hold of their child, as helpful in promoting a positive trajectory (Pérez‐Duarte Mendiola 2024). The findings show how parents and caregivers can be a powerful force in catalysing and challenging healthcare professionals to adopt a child‐centred approach. Unfortunately, this often came because of negative experiences and recognising the importance of their child having a positive experience. Ultimately, there is a need for professionals, parents and caregivers, and children to collaboratively discuss and plan for a child‐centred procedure.

### 6.1 Strengths and Limitations

A strength of the study is that recruitment and data collection methods allowed for the inclusion of participants across cultural and geographic contexts, enhancing transferability. Another strength of the study and sample is the diverse nature of the parent or caregiver's child and their health experiences, with many identified as neurodivergent or with a disability or long‐term health condition.

However, the survey relied on retrospective self‐report, which may be affected by recall, time elapsed since the event, and the emotional intensity of the experience. The written format may have excluded parents and caregivers with literacy, language, or time constraints, and the one‐off data collection only captured experiences at a single point in time. Differences in recruitment and response context between countries had the potential to introduce selection bias. In the UK and NZ, where recruitment was via social media, parents and caregivers with particularly strong experiences (positive or negative) may have been more likely to participate. In Australia, parents and caregivers were approached to participate while in hospital with their child; therefore, their experiences may have been more diverse (positive, negative, or in between). As participation was anonymous, member checking did not occur, and parents were not consulted as part of the analysis process.

As an international study, there were cultural limitations. In NZ specifically, the survey methodology reflects Western research paradigms that may not align with Māori and Pacific worldviews, particularly the emphasis on oral storytelling traditions and collective whānau/aiga perspectives, rather than individual parent accounts. Conducting the survey in English may have disadvantaged parents and caregivers more comfortable in te reo Māori, Pacific languages, or other languages. Similar cultural and linguistic barriers may have affected the participation of diverse ethnic communities across all three countries.

### 6.2 Recommendations for Further Research

This study provides an important foundation for understanding how procedural journeys can unfold and the impact these experiences can have on parents, caregivers, and children. Several directions for future research emerge from the findings.

The retrospective design of this study captured parent and caregiver accounts of a procedure occurring within the last 2 years. Prospective longitudinal research is needed to track children's procedural encounters over time, examining how early experiences of distress and restraint shape subsequent healthcare engagement, fear responses, and longer‐term psychological wellbeing. This prospective design should purposefully sample neurodivergent and intellectually disabled children.

This study captures the parent's perspective, and valuable companion lines of research would gather healthcare professional and child accounts of the same procedural experiences. Exploring alignment or misalignment between parent, professional and child perceptions of the same event would have significant implications for training and reflective practice.

Finally, this study was conducted across three high income predominantly English‐speaking countries with relatively well‐developed children's rights agendas. International replication and cross‐cultural adaptation studies would extend the transferability of these findings and support the development of context‐sensitive guidance.

### 6.3 Implications for Policy and Practice

The parents' accounts of procedural journeys show that unplanned reactive approaches and the use of restraint as default can be a primary driver for distress and traumatic outcomes for both children and their parents/caregivers. This implies that healthcare organisations should mandate a brief procedural planning pause before every non‐urgent procedure involving a child. Policy and practice should require that the role of a parent or caregiver be discussed and negotiated within these pauses to determine whether parents and caregivers will act a comforters, a supportive holders or an observers, rather than making assumptions. As key coordinators of care, nurses are uniquely positioned to champion these pauses, fostering a collaborative approach between parents and the whole healthcare team. Parents and caregivers require information and education to know they have the right to challenge professionals and request a pause or stop a procedure if they are concerned about their child's distress. Health systems and regulatory bodies should formally adopt or align practice with rights‐based standards such as iSUPPORT (Bray et al. 2023) as core principles to underpin child‐centred practice.

## 7 Conclusion

This international qualitative study provides detailed insight into how parents and caregivers experience the restraint of their child during healthcare procedures and the reported short‐ and long‐term impacts of these events. The four procedural journeys identified that unravelling restraint, inevitable restraint led by professionals, inevitable restraint led by parents and caregivers, and child‐centred holding reflect the complexity and variability of how procedures can unfold in practice. It is important to acknowledge that not all uses of restraint are equivalent in their origins or outcomes. Parents' and caregivers' accounts reveal that when procedures unfold without planning, communication, or responsiveness to a child's distress, experiences can escalate rapidly, resulting in fear, loss of trust, and enduring feelings of guilt, regret, and trauma for both the child and parent. In contrast, preparation, collaboration, and a willingness to pause and adapt in response to a child being upset were described as supporting more positive experiences.

This study advances understanding by demonstrating the reported short‐ and longer‐term impact that procedural distress and restraint can have on children and their parents/caregivers. The findings emphasise the importance of parents/caregivers feeling able to challenge professionals and collaboratively find a more child‐centred approach to procedural care. Children's nurses are uniquely positioned to facilitate this shift by championing these collaborative child‐centred practices and acting as key advocates for children's positive procedural experiences.

## Funding

The authors have nothing to report.

## Conflicts of Interest

The authors declare no conflicts of interest.

## Supporting information


**Data S1:** Supporting Information.

## Data Availability

Research data are not shared.
